# The Lungfish Transcriptome: A Glimpse into Molecular Evolution Events at the Transition from Water to Land

**DOI:** 10.1038/srep21571

**Published:** 2016-02-24

**Authors:** Maria Assunta Biscotti, Marco Gerdol, Adriana Canapa, Mariko Forconi, Ettore Olmo, Alberto Pallavicini, Marco Barucca, Manfred Schartl

**Affiliations:** 1 Dipartimento di Scienze della Vita e dell’Ambiente, Università Politecnica delle Marche, Via Brecce Bianche, 60131, Ancona, Italy; 2 Dipartimento di Scienze della Vita, Università di Trieste, Via Licio Giorgeri 5, 34127, Trieste, Italy; 3 Department Physiological Chemistry, Biocenter, University of Würzburg, 97074 Würzburg and Comprehensive Cancer Center Mainfranken, University Clinic Würzburg, 97078 Würzburg, Germany

## Abstract

Lungfish and coelacanths are the only living sarcopterygian fish. The phylogenetic relationship of lungfish to the last common ancestor of tetrapods and their close morphological similarity to their fossil ancestors make this species uniquely interesting. However their genome size, the largest among vertebrates, is hampering the generation of a whole genome sequence. To provide a partial solution to the problem, a high-coverage lungfish reference transcriptome was generated and assembled. The present findings indicate that lungfish, not coelacanths, are the closest relatives to land-adapted vertebrates. Whereas protein-coding genes evolve at a very slow rate, possibly reflecting a “living fossil” status, transposable elements appear to be active and show high diversity, suggesting a role for them in the remarkable expansion of the lungfish genome. Analyses of single genes and gene families documented changes connected to the water to land transition and demonstrated the value of the lungfish reference transcriptome for comparative studies of vertebrate evolution.

Lungfish, discovered over 150 years ago^1^, are a group of sarcopterygian fish, characterized by the presence of lungs and lobed fins. Although, like coelacanths, lungfish were abundant and widespread in the Devonian, only six species of Dipnoi, all living in freshwater habitats, are extant: the Australian lungfish *Neoceratodus forsteri*, which displays the most primitive characters; the South American lungfish *Lepidosiren paradoxa*; and four African species belonging to the genus *Protopterus*, which includes *P. aethiopicus*, *P. amphibius*, *P. dolloi*, and *P. annectens*. Similar to coelacanths, lungfish share several morphological characters with their fossil ancestors and are considered as “living fossils”. Lungfish are of great interest to evolutionary biologists, because they can provide key information on the pre-adaptations, changes in body plan and physiology, and molecular changes that enabled the transition from aquatic to land life. Coelacanths and lungfish can shed light on the last common ancestor of tetrapods and fish and on the scenarios that led to the evolution of land vertebrates.

The phylogenetic relationships among lungfish and the other Sarcopterygii (coelacanths and tetrapods) have long been debated. The three taxa diverged in the early Devonian (380–400 MYA)^2^, in a narrow time window of only twenty million years. Recently, sequencing of the whole coelacanth genome and phylogenomic analysis have led to the conclusion that Dipnoi, not *Latimeria*, are the closest extant relatives to the tetrapod lineage^3^, placing Dipnoi in a unique phylogenetic position to provide information on sarcopterygian evolution.

Genome sequencing provides comprehensive information on the molecular basis of the biology of an organism. Unfortunately, Dipnoi have the largest genomes of all vertebrates. Their DNA content (C-value) is estimated to range from 40 to over 100 pg/cell^4^, compared with only 3.5 pg of humans. Despite the outstanding possibilities offered by current technologies, the sequencing, assembly, and annotation of such large genomes still present insurmountable technical barriers. The fact that a large fraction of the lungfish genome may consist of repetitive DNA, transposable elements (TEs)^5^, and duplications further complicates whole genome analysis.

Until a technology that overcomes these obstacles becomes available, our group set out to generate a lungfish reference transcriptome based on *P. annectens* that can be used by the scientific community to approach a variety of biological issues. The reference transcriptome can provide key information on protein-coding genes, which are a major area of functional genomic research, enable comparison of genes and gene families to those of other organisms, and allow genome-wide search for selection signatures. For any gene of interest, orthologous and paralogous lungfish genes can be added to gene trees; moreover, novel gene gains and losses in the dipnoan lineage can be partially assessed.

According to annotation statistics, our newly assembled transcriptome provides a good representation of the protein-coding fraction of the lungfish genome. The transcriptome was used to gain evolutionary and phylogenetic insights into basal sarcopterygian genomics. The present work confirmed lungfish as the closest living relatives of tetrapods, and highlighted a slow evolutionary rate of protein-coding genes. The high diversity and activity of TEs is consistent with a potential role for these sequences as driving forces in shaping genome size. Examination of genes likely to be involved in adaptation to land disclosed an expansion of those encoding lung surfactant proteins and changes in those involved in fin to limb transition and nitrogen excretion in non-aquatic environments.

## Results

### Assembly and annotation of the *P. annectens* transcriptome and generation of reference sequences

The Illumina sequencing procedure generated 677,978,082 raw nucleotide paired-end reads from male and female brain, liver, and gonads (Supplementary Table S1). To ensure greater sequencing coverage, the *P. annectens* Illumina sequencing data obtained for liver, kidney and brain in a previous investigation^3^ were added to the current dataset, yielding a total of 812,328,905 paired-end reads (71.17 Gb of sequence data). *De novo* transcriptome assembly generated 177,760 contigs (for read and assembly statistics, see Supplementary Tables S2 and S3). Clustering of the assembled contigs based on a 90% sequence identity threshold showed limited redundancy in the assembled transcriptome.

The TransDecoder annotation of a set of 74,318 well-supported contigs (displaying a sequencing coverage >10 X), allowed identification of 27,274 protein-coding contigs; of these, 18,125 showed a significant BLAST match in the UniProtKB/Swiss-Prot database. GO cellular component (15,719 contigs), GO biological process (14,008), GO molecular function (14,312), and eggNOG (8,433) annotations were inferred from these data. Moreover, InterPro analysis of the 27,274 predicted proteins allowed identifying 18,474 contigs containing conserved domains.

The integrity of the annotated protein-coding transcripts was assessed with a modified Ortholog Hit Ratio method^6^,^7^, which indicated that ~65% of the assembled transcripts were full-length compared with the orthologous genes of the other basal aquatic sarcopterygian *L. chalumnae* (Supplementary Fig. S1). 15,399 out of the 23,601 genes annotated in *L. chalumnae* (~65%) were scored as reciprocal best hits (likely orthologs) and 22,927 (~97%) constituted a significant match in the lungfish transcriptome. For comparison, very similar rates (in ~63% reciprocal best hits and ~94% significant matches) were obtained between *D. rerio* and *O. latipes*, which belong to lineages that split from one another approximately 300 million years ago.

**Figure 1:**
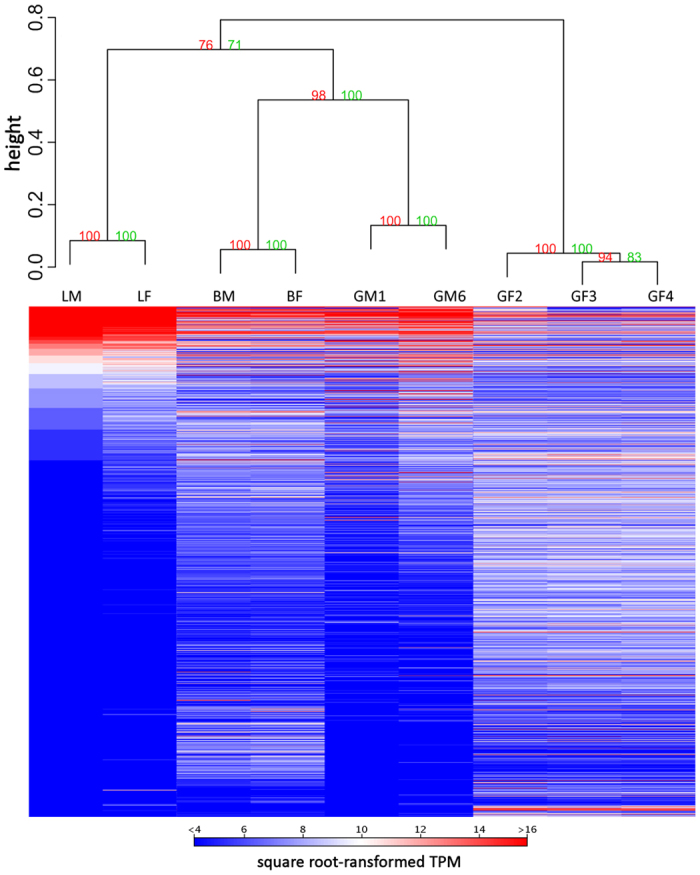
Hierarchical clustering of the lungfish tissue samples analysed based on gene expression data (see Supplementary Table S1 for details on tissue samples). Square-root transformed TPM values were employed for hierarchical cluster analysis using an average method and correlation-based dissimilarity matrix. Numbers on nodes indicate bootstrap support values. To maximize the visualization of heat map data, only transcripts with a minimum transformed TPM value of 4 in at least one sample are displayed.

The gene expression profiles of the 9 samples showed a well-supported tissue-based clustering (Fig. 1). Principal Component Analysis (PCA) highlighted a very high similarity of male and female brains (Supplementary Fig. S2), whereas a slight difference was noted between male and female liver. Such dimorphism has also been reported in other fish, and reflects differences in metabolism and sex-specific functions, like the production of yolk protein in females^8^,^9^. A more marked difference, detected between the two male gonad samples, can be explained by the fact that they were collected from two individuals at different stages of sexual maturation.

### Implications of the lungfish mobilome for the transcriptome

The analysis carried out to identify transcribed repetitive elements in the *P. annectens* sequences disclosed that 7.20% of the assembled contigs harboured at least one repeat, and that 2.47% of sequenced bases matched a RepeatMasker entry (Table 1). LINEs (45.66%) accounted for the largest number of hits, followed by DNA transposons (32.39%), and SINEs (13.75%); LTRs (7.93%) were the least represented elements (Supplementary Fig. S3). However, the abundance of some TE types in the transcriptome may not necessarily reflect their activity.

**Figure 2:**
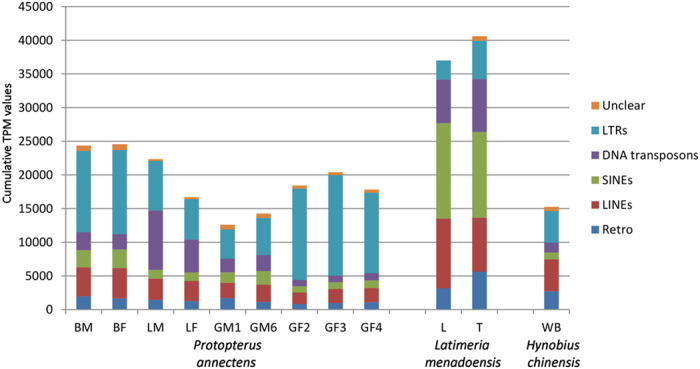
Comparative transcription activity of the main TE classes in the *P. annectens*, *L. menadoensis*, and *H. chinensis* transcriptomes. Activity is expressed as cumulative TPM values of the elements pertaining to each class. TPM values were calculated on a set of 2,111 evolutionarily conserved genes, as detailed in Materials and Methods. Retro: retroelements that could not be classified with certainty as LTRs or non-LTRs. L: liver; T: testis; WB: whole body.

The tissue where TEs were most active was the brain (both sexes) and the one where they were least active was the immature male gonad (Fig. 2). In all tissues the largest fraction was represented by LTRs (mainly DIRS), followed by LINEs (mainly L2, CR1 and Penelope) (data not shown); however, DNA transposons also appeared to be highly active in liver.

Overall, TE activity in lungfish tissues was comparable to or slightly higher than that of newt, and lower (by 40–70%, depending on tissue) than that determined in coelacanth liver and testis (Fig. 2). Lungfish and newt displayed a predominant activity of LINEs and LTRs, whereas SINEs were the most active TEs in the Indonesian coelacanth.

**Figure 3:**
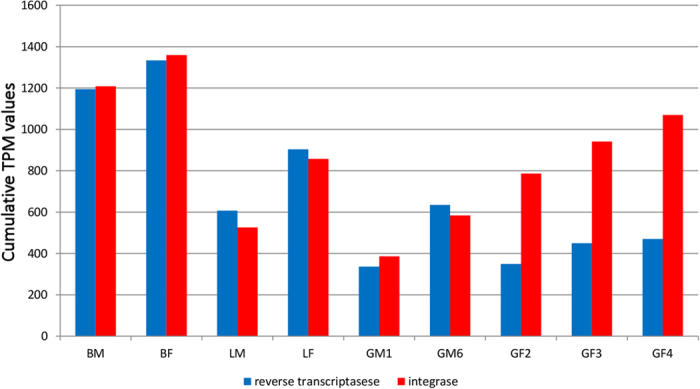
Cumulative expression of reverse transcriptase and integrase domains containing transcripts in the *P. annectens* tissues analysed.

The higher LTR and LINE activity in lungfish was confirmed by the high abundance of two InterPro protein domain annotations: the reverse transcriptase (IPR000477) and the integrase/recombinase N-terminal (IPR023109) domain. The former domain is found in the transcripts of all autonomous retroelements (LINEs and LTRs), whereas the integrase domain is confined to Gypsy and ERV LTR elements. Expression analysis of the transcripts annotated with these two domains demonstrated a very similar activity of reverse transcriptases and integrases in all tissues except female gonad, where integrases were far more expressed (Fig. 3). The different patterns can be explained by a higher expression of LTRs compared with LINEs.

The analysis directed at identifying the expansion of TE families in lungfish compared with representative species of Actinopterygii and tetrapods by InterPro domain annotation revealed 14 over-represented domains compared with Actinopterygii (Supplementary Table S4). The limited redundancy of the assembled transcriptome and the application of stringent filtering sequence similarity criteria make it highly unlikely that expansion events were related to splicing variants, inter-individual polymorphisms, or sequencing errors. Of the 14 over-represented gene families, 9 were also significantly expanded compared with tetrapods. Most over-represented domains are linked to transposon activity (integrase, reverse transcriptase, and endonuclease), indicating high TE diversity in lungfish.

### Slow evolution and phylogenetic proximity to tetrapods

**Figure 4:**
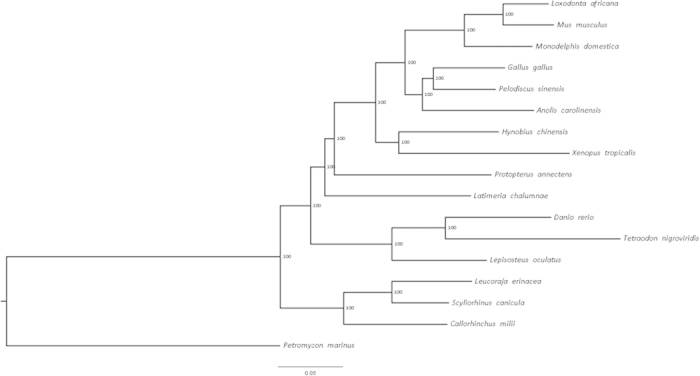
Phylogenetic tree of representative vertebrates based on the alignment of 226 evolutionarily conserved genes (see list in the Materials and Methods section).

Whether lungfish or coelacanths are the closest living relatives to tetrapods is a long-debated issue^10^. Phylogenetic inference after the completion of coelacanth genome sequencing lent support to the former notion. Further support comes from the present independent phylogenomic analysis of a solid dataset of 226 concatenated protein sequences, encoded by orthologous genes conserved in all vertebrates and accounting for 59,951 informative amino acid positions, which identified both the lungfish/tetrapod and the coelacanth/lungfish + tetrapod nodes (Fig. 4).

Besides sharing a key position in the tree of life with respect to the water to land/air transition, lungfish and coelacanths also have several common morphological features that have remained basically unchanged compared with the prehistoric fossil record. This raises the question whether such a high degree of morphological conservation is accompanied by a slow rate of genomic evolution. Application of Tajima’s Relative Rate Test^11^ to the concatenated sequence set using the three chondrichthyan species as the outgroups to find evidence of significant differences in the rate of molecular evolution between lungfish (outgroup I) and the other vertebrates (outgroup II) disclosed that the lungfish genome is indeed evolving more slowly than those of mammals/marsupials, bony fishes (including the basal branch of Lepisosteiformes), and most tetrapods (Supplementary Table S5). On the other hand, lungfish protein-coding genes appear to share a similar rate of evolution with other tetrapods, including chicken, the Chinese soft shell turtle and, curiously, the Chinese salamander, another organism with a giant genome. Moreover, differences between lungfish and coelacanth were not significant.

### Evolutionary retentions and innovations

#### Fin to limb

**Figure 5:**
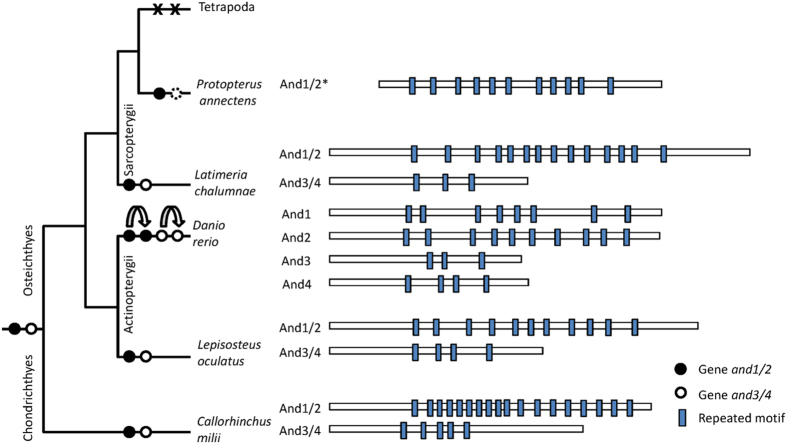
Actinodin (*and*) gene evolution and protein structures. Blue boxes: Repeated motif C(N/D)PXXDPXC; black circles: *and1/2*; white circles: *and3/4*; dashed circle: putative missing gene; X: gene loss; curved arrow: duplication event; *incomplete sequence at N-ter. Boxes representing proteins reported in scale.

Actinotrichia are skeletal elements in the fins of Actinopterygian (ray-finned) fish and are homologous to the ceratotrichia found in Chondrichthyes. Both are made of elastoidin, a mix of collagenous and non-collagenous proteins^12^. In zebrafish, the actinodin proteins (And) of the non-collagen fraction are essential components of actinotrichia^13^. Outside ray-finned fish, sequences encoding these proteins have been identified in the shark *C. milii*^13^ and in *L. chalumnae*^3^. Actinotrichia of greatly reduced size can still be identified in the fins of *L. chalumnae*^14^. The fin rays of lungfish, or camptotrichia, lack actinotrichia and show a unique structure and organization^15^. Actinodin genes have never been detected in any of the tetrapod genomes sequenced to date. This constitutes a notable difference in the otherwise very similar early development of tetrapod limb and fish fin buds. Since loss of these genes during evolution may be related to the water to land transition, *and* genes were sought in *P. annectens*. The result was a single partial *and* transcript (incomplete at the 5′ end, likely due to low expression in the tissues analysed). Analysis of the amino acid sequence disclosed 11 repeats of a motif constituted by the 9 amino acids C(N/D)PXXDPXC, characteristic of these proteins (Fig. 5). Phylogenetic analysis (Supplementary Fig. S4) of And sequences from representative gnathostomes attributed the lungfish sequence to the And1/2 group. The sequence obtained in the present study allows to trace the evolutionary history of the *and* gene family. The gnathostome ancestor had two genes, *and1/2* and *and3/4*; in the teleost ancestor they underwent a duplication event that led to four paralogous genes as a result of teleost genome duplication (Fig. 5). The *and* gene homologues detected in lungfish puts forward the loss of *and1/2* to the tetrapod ancestor. However, the disappearance of the *and3/4* gene remains unclear. The gene is still found in coelacanths, but not in the *P. annectens* transcriptome. Although its absence could merely be related to lack of expression in the tissues analysed, which did not include RNA-seq from fin tissue, it is possible that *and3/4* was lost in the common ancestor of lungfish and tetrapods after the split from coelacanths.

The lack of evidence for pseudogenization of the *P. annectens and1/2* gene suggests that it may have been recruited for the formation of the camptotrichia, which support the fins in Dipnoi.

*Rapunzel* (*rpz*) is a recently identified zebrafish gene family, comprising five paralogous genes, that is involved in the number and size of fin ray segments. Although their specific functions are not yet entirely clear, functional redundancy is unlikely^16^. Like *and* genes, *rpz* homologous genes have not been described in the tetrapod clade.

Ten non-redundant sequences pertaining to the *rpz* gene family were identified in the lungfish transcriptome. The finding might be related to the morphology of lungfish appendages, which present numerous serially repeated elements^17^. The phylogenetic analysis highlighted species-specific duplication events, with four sequences assigned to the *rpz1/rpz2* and six assigned to the *rpz3/rpz4/rpz5* subgroups (Supplementary Fig. S5). Detection of these genes in lungfish and Actinopterygii suggests their presence in the common ancestor of Actinopterygii and Sarcopterygii, whereas their absence in *Latimeria* and tetrapods would indicate two independent, lineage-specific, gene loss events.

#### Pulmonary surfactants

The transition from the aquatic to the terrestrial lifestyle also included profound changes in the respiratory system. African lungfish are obligatory air-breathers and possess paired lungs extending throughout the length of the body cavity. Although the origin of vertebrate lungs is still debated, the homology between the tetrapod lung and the fish swimbladder has recently been evidenced^18^. Pulmonary surfactant is a complex lipoprotein mixture secreted by lung alveolar cells to reduce the surface tension of fluids. Four types of proteins associated with pulmonary surfactants (SFTP), A, B, C, and D, are important components of human surfactants^19^. The evolutionarily related SFTPA and D are collectins involved in the innate immune defence of the lung. SFTPB and C are small hydrophobic proteins produced from high molecular weight precursors.

To date, SFTPA, C, and D have been annotated only in tetrapods (with a putative SFTPD being detected in coelacanths in the present study), whereas SFTPB is evolutionarily conserved across vertebrates. However, five putative SFTPA/D/MBL and three putative SFTPC sequences, but not SFTPB, were retrieved in the transcriptome of *P. annectens*. The high level of sequence similarity, and, for the putative SFTPC, the presence of the predicted transmembrane region characteristic of the mature peptide (Supplementary Fig. S6A) support the relatedness of these sequences to the pulmonary surfactant protein fraction. The phylogenetic analysis (Supplementary Fig. S7) assigns the sequences identified in *P. annectens* to the A/D/MBL group, but it does not link them to individual genes. These genes are found in an unresolved position between *SFTPA*/*D* genes and their evolutionary closest neighbours *MBL* (Mannose binding lectin) and *HBL4* (Hexose binding lectin) genes, which are involved respectively in mannose binding and the innate immune response in mammals and teleosts. The *A*, *D*, and *MBL* genes are organized in clusters^20^, probably as a result of gene duplications. Whereas a single gene of this group is found in the *L. chalumnae* genome, the five sequences identified in the lungfish transcriptome suggest a series of lineage-specific duplication events. The closer proximity of the lungfish sequences to the tetrapod *SFTPA* genes (though not supported by high values of posterior probability) agrees with the evolutionary scenario hypothesized by Crouch^21^, where the *SFTPD* and *MBL* genes would have a common origin from *SFTPA*. Thus, the duplication events that led to the current *SFTPA*, *SFTPD* and *MBL* genes in the tetrapod lineage likely occurred after the split from lungfish. The expansion of this gene family, which occurred independently in the two lineages, may be connected to new respiratory functions related to the water to land transition.

The present phylogenetic analysis suggests that *SFTPC* originated in the common ancestor of Sarcopterygii and subsequently underwent lineage-specific duplication in lungfish (Supplementary Fig. S6B). However, the three lungfish *SFTPC* genes are clearly divergent from the single *SFTPC* of tetrapods, suggesting a functional specialization that may have occurred in this lineage during the conquest of land.

#### Urea production

Lungfish are facultative ureotelic organisms: i.e. they are mostly ammoniotelic in the water environment, producing ammonia that is excreted through branchial and cutaneous epithelia. In conditions of limited water availability, such as aestivation, they switch to ureotely, converting ammonia to urea, which is stored in the body^22^. Urea excretion, which is widespread among and almost exclusive to amphibians (except water-residing larval stages), some turtles, and mammals, has generated the hypothesis that the switch from ammonia to urea as the main nitrogenous waste product was a key step in the transition from water to land. Urea is produced by two pathways, purine catabolism and the urea cycle (Supplementary Fig. S8). Genes coding for all the enzymes involved in these pathways were retrieved in the *P. annectens* transcriptome.

As regards urea cycle genes, it has been noted^3^ that the rate-limiting enzyme carbamoyl phosphate synthase I is positively selected for in lungfish and coelacanths, pointing at the importance of this metabolic cycle in the adaptation to life on land. In addition, purifying selection was found for all the enzymes involved in the purine catabolism pathway (Supplementary Table S6). Two genes encoding *HIUase* isoforms are found in *Latimeria*, whereas a single gene was identified in the lungfish transcriptome, as in all tetrapods. This confirms the notion^23^ that the second *HIUase* gene is the result of a coelacanth-specific duplication with sub-functionalization of a liver and a testis isoform.

## Discussion

The reference transcriptome of the West African lungfish, generated in the present study, is representative of the Dipnoan clade and provides for the first time extensive sequence information on the species. Even though transcriptomes obtained from RNA-seq data may lack transcripts that are not expressed in the source material, the reference transcriptomes of eukaryotes, whose genome has not been sequenced, have nonetheless been found to mirror the exome quite closely^24^. The present transcriptome annotation documented more than 20,000 protein-coding genes with considerable coverage (>10 X), which likely account for a large fraction of those encoded in the lungfish genome. The transcriptome will provide a useful resource until a full genome sequence becomes available, since transcript sequences can be employed in molecular phylogenetic and genome evolution studies. Further analyses will supply key information on several issues, including molecular processes that were important in the water to land transition and on adaptations of the lungfish themselves, which at least morphologically have remained quite similar to their ancestors that lived about 400 Mya.

Even though genome size is highly variable in eukaryotes, lungfish are almost unique among vertebrates, together with some amphibians, in having a very large genome. Such size has a number of effects, for example on nuclear volume, cell size, duration of the cell cycle, metabolic rate, and extinction rate^25^.

Even though a transcriptome does not represent the whole genome, the lungfish transcriptome can nonetheless help explain the large genome size. One possible explanation may be several rounds of genome duplication. Even though earlier karyotype studies do not support recent polyploidization events except in *P. dolloi*^26^, very ancient whole genome duplication (WGD) events do not necessarily involve a higher chromosome number, since they are generally followed by a genome reduction due to remodelling phenomena, as seen for instance in angiosperms and teleost fish^27^,^28^,^29^,^30^. Analysis of the lungfish transcriptome suggested the lack of recent lineage-specific polyploidization. In terms of number of assembled transcripts its size is comparable to that of other recent Illumina transcriptomes of diploid piscine species^7^,^31^, or of other larger vertebrates such as *Ambystoma tigrinum*^32^. The number of protein-coding genes is in the range predicted for the complete genomes of other vertebrates. Moreover, there is evidence of expansion only for a limited number of gene families, which, as discussed below, are those mostly closely linked to TE activity (Supplementary Table S4).

Genome size increases may also be related to TE accumulation. A study of a very small fraction (~1%) of the genome of the Australian lungfish *N. forsteri*^33^ suggested that 40% of it consists of TEs, mainly non-LTR elements like LINEs (CR1 and L2). The authors argue that the *N. forsteri* genome is the result of massive TE amplification followed by a long period with very low rates of TE removal and some ongoing TE activity. The remaining portion includes old, highly divergent, and probably inactive TE copies^5^,^33^. Similarly, the increase in the salamander genome size has been associated with the expansion of TEs, particularly LTRs^34^.

We found a greater variety of protein domains typical of TEs, including integrases, reverse transcriptases, and endonucleases, in the transcriptome of *P. annectens* compared with other vertebrates, suggesting that their explosion may have contributed to the current genome size. The presence of TEs in the transcriptome data (7.20% of contigs assembled compared with 11.17% in *Latimeria*) and their non elevated expression level (~0.5–2% compared with a set of evolutionarily conserved genes used in the analysis) suggest that the TE expansion occurred in the very distant past, and that the copies found in the present genome may be old and largely inactive. This is consistent with the hypothesis^5^ that waves of transposition occurred about 300 Mya, followed by TE inactivation in lungfish and salamanders. This view might be in line with the comparable TE expression level found in *Hynobius* and lungfish in the present study.

Despite the greater number of TEs found in the *P. annectens* contigs, and in agreement with the finding that LINEs are highly represented in the *N. forsteri* genome^33^, the most highly expressed class in the lungfish transcriptome are LTR elements. Although in the salamander genome LTR elements are predominant^34^, LINE elements were slightly more active in the transcriptome (see Fig. 2). In both species, this might be explained by an ancient expansion that led to the predominance of some TE classes, whereas in the current genome their ongoing silencing makes the expanded TE classes less active and thus less abundant in the transcriptome^35^.

Two further gene family expansion events involved Krüppel-associated box (IPR001909) and the GIY-YIG nuclease superfamily (IPR027299, IPR000305). The Krüppel-associated box zinc finger family, which to date has been described as tetrapod-specific, has been hypothesized to play an important role in silencing endogenous retroviruses^36^. Its strong representation in lungfish (but not in coelacanth) appears to reflect an innovation at the base of the sarcopterygian lineage. The GIY-YIG nuclease superfamily is involved in genome stability, including DNA repair and recombination, movement of non-LTR retrotransposons, maintenance of gene copy number, and restriction of incoming foreign DNA^37^. The expansion of this family may be interpreted as a genomic defence mechanism against the threat of a spreading mobilome.

The transition from water to land required massive changes in physiology and body plan, some of which are already obvious in the amphibious lungfish. A major change was the evolution of paired fins to the tetrapod limbs, while the fin rays disappeared and were replaced by digits. A major structural component of the terminal fin rays, the actinodin proteins, are encoded by four genes (two paralogous groups) in fish, that are not found in tetrapods. The present work demonstrates that whereas the coelacanth retains the two ancestral *and* genes (*and1/2* and *and3/4*, one previously not annotated), only the *and1/2* sequence is found in the lungfish transcriptome. Even though this requires confirmation at the genomic level, it may reflect the progressive elimination of genes that have become unnecessary.

An additional finding was that *rapunzel* family genes- which are involved in regulating zebrafish fin ray segment size and number and are not extant in *Latimeria* and tetrapods -are retained in lungfish, as reflected by the amplification of members of certain branches of this family and the loss of others. The finding may be related to specific lungfish features, maybe in connection with the formation of camptotrichian fin rays. The *rapunzel* genes identified in lungfish suggest that several changes connected with tetrapod limb formation occurred in Sarcopterygii after the split of lungfish from tetrapods, most likely after the transition to land.

Another key process in land adaptation was the development of air-breathing lungs. Several proteins in the tetrapod lung are major components of the surfactant, which plays a role in gas exchange and immune defence, but are not found in Actinopterygian fish. In addition, the *SFTPA* gene has not been identified in the coelacanth genome. The detection of surfactant proteins in lungfish underscores the evolutionary need for such genes for the development of air-breathing lungs. A lineage-specific gene family expansion occurred in *Protopterus* and resulted in an even greater number of *SFTP* genes than in tetrapods, which may have been critical for the lungfish amphibious lifestyle.

As regards the two urea producing pathways, which address the problem of nitrogen excretion in situations of limited water availability, the only gene under selection was the rate-limiting enzyme of the urea cycle; all the other genes involved in the cycle and in the alternative pathway displayed purifying selection. Clearly, a single change was sufficient to adapt the excretion metabolism.

The long-debated question of which living fish species is most closely related to the common ancestor of all tetrapods was settled when a large coelacanth gene dataset became available in 2013^3^. The present work confirms that lungfish, not *Latimeria*, are the closest extant relatives of tetrapods. The similar branch length of the lungfish/tetrapod and coelacanth/lungfish + tetrapod nodes suggest that the three lineages diverged over a relatively short period. This may also partly explain why previous studies based on smaller sequence datasets concluded that the relationship among the three groups was an “irresolvable trichotomy”^38^.

The morphological similarity of lungfish and coelacanths to their fossil ancestors has prompted their designation as “living fossils”. Analysis of the coelacanth genome has demonstrated that the *Latimeria* protein-coding genes are evolving at a significantly slower rate than in chicken, mammals and, importantly, lungfish^3^. However, analysis of a sound dataset of evolutionarily conserved genes, obtained in this study, did not yield definite evidence for a faster evolution of lungfish compared with coelacanth protein-coding genes. The slow rate of evolution of *Latimeria* protein-coding genes has been hypothesized to relate to low-level predation in the deep marine habitat, hence to a limited need for adaptation throughout the evolutionary history of the species. The extant lungfish species definitely faced demanding conditions in the various freshwater habitats, including estivation in dry periods. A closer look at the individual genes that are predicted to be involved in lungfish adaptations, and the identification of those showing high levels of positive selection may provide insights into the selection pressures that shaped the lungfish genome and explain why the vast majority of protein-coding genes evolve so slowly.

In contrast, another relevant fraction of the large lungfish genome represented in the transcriptome, the transposable elements, is not any different from fast evolving genomes. The lungfish transcriptome contains sequences that represent a large diversity of transposable elements. Thus, like in the coelacanth, the protein-coding fraction of the genome appears to evolve rather slowly while TE fraction does not appear to be of low plasticity or stasis.

This first set of analyses of the annotated transcriptome of the lungfish *P. annectens* demonstrates its value as a resource for future studies of the gene and genome evolution of vertebrates and sheds some light on the processes associated with the water to land transition.

## Material and Methods

### Tissue sampling and RNA extraction

Male and female *P. annectens* juveniles were obtained from a local fish importer. All animals were kept and sampled in accordance with the applicable EU and national German legislation governing animal experimentation, in particular all experimental protocols were approved through an authorization (568/300-1870/13) of the Veterinary Office of the District Government of Lower Franconia, Germany, in accordance with the German Animal Protection Law (TierSchG). Species identity was confirmed by partial sequencing of the 16 S gene essentially as described by Tokita *et al.*^39^, except that the annealing temperature was set to 56 °C. Brain, liver and gonads were employed for total RNA isolation using either the RNeasy Midi Kit with DNase on-column digestion (Qiagen, Germany) or the Trizol protocol (TRIzol^®^ Reagent, Life Technologies, USA), followed by DNase digestion of 30 μg RNA with 15 U DNase I (ThermoScientific, USA) in a total volume of 100 μl containing 100 U RNase Inhibitor (RiboLock, ThermoScientific, USA) for 1 h at 37 °C. This step was followed by phenol/chloroform extraction or DNase in-solution digestion and column purification using RNeasy MinElute Cleanup Kit (Qiagen, Germany). One immature and one mature testis, three ovaries, and a brain and a liver each from a male and a female specimen (Supplementary Table S1) were processed for RNA sequencing. RNA integrity number (RIN) values ranged from 7.4 to 9.1.

### Sequencing

Transcriptome sequencing was performed on an Illumina HiSeq2000 platform at BGI Tech (Hong Kong). cDNA libraries were prepared with the TruSeq kit according to the manufacturer’s instructions and were subjected to 90 cycles of paired-end sequencing (one sample/lane).

### *De novo* transcriptome assembly, annotation and quality assessment

The raw sequencing output was imported into a CLC Genomics Workbench 7.0.3 platform (CLC bio, Denmark) and trimmed using a base caller quality threshold of 0.05. Illumina sequencing adapters were removed; reads shorter than 75 base pairs after trimming were removed prior to assembly. Transcriptome assembly was performed with the *de novo* assembly tool of the CLC Genomics Workbench; both novel sequencing reads and those previously generated for the same lungfish species in the framework of the coelacanth genome project^3^ (Bioproject accession: PRJNA164839, tissues: brain, kidney, liver) were used as the inputs. *Word size* and *bubble siz*e parameters were automatically estimated; scaffolding was allowed and the minimum contig length was set to 250 nucleotides.

Contigs resulting from mitochondrial and nuclear RNAs were detected by BLASTn^40^, based on a positive match (e-value threshold, 1 × 10^−10^) with the complete *P. annectens* mitochondrial DNA sequence^41^ and with *Xenopus laevis* 18 S/5.8 S/28 S precursor rRNA (accession ID: X59734.1), respectively. The integrity of the assembled protein-coding transcripts was assessed with the Ortholog Hit Ratio Method^6^, based on comparison with the orthologous genes found in the fully sequenced genome of *L. chalumnae*^3^, using BLASTx and an e-value cut-off of 1 × 10^−5^. Since this method may underestimate integrity, due to inter-species divergence, analyses were confined to genes displaying high levels of identity between the species (>90%), as suggested by Pallavicini and colleagues^7^. Transcriptome redundancy was estimated by CD-HIT-EST v.4.6.1^42^, using a 90% sequence identity threshold.

Paired-end reads were then re-mapped on the assembled contigs to calculate coverage. A subset of highly reliable contigs (achieving an average coverage >10 X) were selected for subsequent annotation and gene expression analyses, to remove low-expression noise and unreliable and fragmented transcripts^7^,^43^,^44^. Annotation was performed with Trinotate r20140708: first, sequences were analysed with TransDecoder (https://transdecoder.github.io) to produce *in silico*-predicted proteins longer than 100 amino acids, which were then subjected to BLASTp analysis^40^ against the UniProtKB/Swiss-Prot database (e-value threshold, 1 × 10^−5^). Significant hits were used to retrieve Gene Ontology (GO) and “evolutionary genealogy of genes non supervised orthologous groups” (eggNOG) annotations for matching contigs^45^,^46^. Predicted proteins were scanned with InterProScan 5^47^, to detect conserved domains^48^ (e-value threshold, 1 × 10^−5^). The 30 most abundant domains are listed in Supplementary Table S7.

Raw RNA-seq data have been deposited at the NCBI Sequence Read Archive (BioProject accession ID: PRJNA282925) and are available upon request.

### Gene expression analysis

Trimmed sequencing reads obtained from each of the 9 tissue samples (Supplementary Table S2) were individually re-mapped to the annotated reference transcriptome using the RNA-seq mapping tool of the CLC Genomics Workbench, setting length and similarity fraction respectively to 0.75 and 0.95, and insertion/deletion/mismatch penalties to 3/3/3.

Gene expression levels were calculated as Transcripts Per Million (TPMs), a measure of RNA abundance that is proportional to the relative molar RNA concentration and efficiently takes into account both transcript length and sequencing depth^48^.

A Baggerly beta-binomial test on proportions was applied to detect genes differentially expressed across samples^49^ in the following comparisons: a) male vs. female liver; b) male vs. female brain; c) mature vs. immature male gonad. The criteria applied to detect differential expression were set at a minimum weighted proportion fold change of ±2 and a Bonferroni-corrected p-value < 0.01. The annotations of differentially expressed genes were used for a hypergeometric test to highlight over-represented InterPro domains and GO and eggNOG terms in each subset. Annotations displaying p-values < 1 × 10^−5^ and values of observed matches >5 were considered as significant over-representations.

Square-root transformed gene expression values were computed with the R (v.3.2.2) package *pvclust*^50^,^51^; hierarchical cluster analysis was conducted with multiscale bootstrap (1,000 replicates) using the average method and a correlation-based dissimilarity matrix as well as PCA analysis with the CLC Genomics Workbench.

### Identification and classification of TEs

First, the whole *de novo* lungfish transcriptome assembly was scanned with RepeatMasker 4.0.3^52^ (http://www.repeatmasker.org) against the vertebrate repeats database Dfam1.2., to determine the number of each TE class that was found in the lungfish sequences. Then, novel repeats were sought with RepeatScout using default parameters^53^ in lungfish, *L. menadoensis*^7^ and *Hynobius chinensis* transcriptomes^54^, which had been re-assembled using the parameters that had been applied for *P. annectens*. False positives were detected and managed as follows: (i) repeats showing a positive BLAStx match (e-value cut-off, 1 × 10^−5^) in *L. chalumnae* and *Xenopus tropicalis* predicted proteomes were removed; (ii) repeats with InterPro domains were removed; (iii) sequences removed in steps (i) and (ii) that contained domains related to TEs (e.g. integrase, reverse transcriptase, etc.) were re-included in the repeat library. Only repeats longer than 100 nt were selected for further analysis, since shorter lengths prevent mapping of RNA-seq reads. The lungfish repeat library built with RepeatScout consisted of 518 items.

The repeat libraries of the three species were analysed with TEClass^55^ to distinguish elements into DNA transposons, LTRs, LINEs, or SINEs; whenever a retroelement could not be classified with certainty as LTR or non-LTR, it was simply designated as a “retroelement”.

The cumulative expression of the two large TE families containing reverse transcriptase (IPR000477) and integrase (IPR023109) domains identified in *P. annectens* were determined by summing the TPM expression values of all transcripts displaying these annotations.

Then TE activity was estimated in lungfish, coelacanth, and newt. Comparing gene expression across species is a major challenge of comparative genomics in the next generation sequencing era, given the scope for systematic error due to different genome and transcriptome size and quality of annotation^56^. In the present case, comparison of TE activity was further complicated by their non-orthologous nature and by redundancy across transcripts. The problem was addressed by adopting a heuristic approach, where the calculation was calibrated on a set of 2,111 unequivocal transcripts (based on tBLASTx reciprocal best hits with an e-value threshold of 1 × 10^−5^) that are orthologous among the three species, as suggested by other studies^57^. In brief, the *per million* scaling factor was computed for each reduced transcriptome using the parameters reported above; then the scaling factor was used to compute the expression of each TE, and the total transcriptional activity of each TE class was calculated in each tissue and species. The process provided an estimate of TE activity relative to a common reference set of orthologous genes.

### Protein domain and gene family expansion analysis

The InterPro annotations of the *P. annectens* contigs were used to identify protein domains and gene families that were likely to have undergone significant expansion in lungfish compared with ray-finned fish and tetrapods. All domains annotated in at least 20 lungfish contigs were included in the analysis. Despite the non-redundant nature of the lungfish transcriptome (see the Results), potential redundancies - i.e. variants generated by alternative splicing, sequencing errors, or allelic polymorphisms - were avoided by removing similar sequences with CD-HIT v.4.6.1^42^, based on a highly stringent similarity threshold (75% identity at the amino acid level). Protein domain annotations for the fully sequenced genomes of the following organisms (from InterPro: http://www.ebi.ac.uk/interpro/) were retrieved and counted: *Danio rerio*, *Astyanax mexicanus*, *Lepisosteus oculatus*, *Gasterosteus aculeatus*, *Takifugu rubripes*, *Tetraodon nigroviridis*, *Oryzias latipes*, *Oreochromis niloticus*, and *Oncorhynchus mykiss* (Actinopterygii); *Homo sapiens*, *Mus musculus*, *Monodelphis domestica*, *Ornithorhynchus anatinus*, *X. tropicalis*, *Anolis carolinensis*, *Pelodiscus sinensis*, *Gallus gallus*, and *Taeniopygia guttata* (Tetrapoda). Significant over-representation of protein domains in lungfish compared with Actinopterygii and Tetrapoda was assessed with one-sided Grubbs test for outliers. The threshold for over-representation - indicating expansion of a gene family - was set p < 0.05.

### Phylogenetic analysis

For each gene family of interest, sequences obtained from the Ensembl and NCBI databases were aligned with ClustalW2 (http://www.ebi.ac.uk/Tools/msa/clustalw2/) using default parameters, and phylogenetic analysis was conducted with MrBayes v.3.1.2^58^. Parameters, model, stationarity, and rooting are reported in the legend to each tree.

### Rate of molecular evolution

A reliable set of orthologous protein-coding genes was identified in 17 chordates and used to infer the rate of molecular evolution in lungfish compared with the following vertebrate species: *A. carolinensis*, *Callorhinchus milii*, *D. rerio*, *G. gallus*, *H. chinensis*, *L. chalumnae*, *Leucoraja erinacea*, *L. oculatus*, *Loxodonta africana*, *M. domestica*, *M. musculus*, *P. sinensis*, *Petromyzon marinus*, *Scyliorhinus canicula*, *T. nigroviridis*, and *X. tropicalis*. Most protein-coding sequences were retrieved from Ensembl (http://www.ensembl.org/index.html); assembled transcripts for the three chondrichthyan species were retrieved from SkateBase^59^ and their coding sequences (CDSs) were predicted with TransDecoder. The same method was used for the *de novo* assembled *H. chinensis* transcriptome.

All CDSs were translated into proteins; then reciprocal BLASTp (e-value threshold, 1 × 10^−50^; only the best hit was considered) allowed identification of a set of 226 genes found in all 17 species with a 1:1 orthology ratio. Protein sequences were aligned with MUSCLE^60^; alignments were refined with Gblocks v.0.91b^61^ to remove divergent regions or missing positions of the alignment due to erroneous gene predictions or to the incomplete nature of the transcriptome datasets used in the comparative analysis.

The resulting sequence datasets were concatenated into a single alignment file comprising 59,951 informative amino acid positions. The file was used to conduct Tajima’s Relative Rate Test of molecular evolutionary rates. The three chondrichthyan species were used separately as the outgroup to test the relative rate of molecular evolution of *P. annectens* proteins (ingroup I) compared with other vertebrates (ingroup II). P-values < 0.05 were considered to be indicative of a significantly slower evolutionary rate.

The same dataset was used for Bayesian phylogenetic analysis with MrBayes^58^, under a General Time Reversible (GTR) substitution model, with a gamma distributed rate of variation among sites and a proportion of invariable sites (GTR + γ + I); this has been described as the best model to fit the data by ProtTest 3^62^. *P. marinus* was used as the outgroup to root the tree.

The ω rates (non‐synonymous/synonymous substitutions) of *P. annectens* and other representative vertebrates for each gene involved in purine catabolism were obtained with KaKs_calculator^63^ according to Goldman and Yang^64^.

## Additional Information

**How to cite this article**: Biscotti, M. A. *et al.* The Lungfish Transcriptome: A Glimpse into Molecular Evolution Events at the Transition from Water to Land. *Sci. Rep.* **6**, 21571; doi: 10.1038/srep21571 (2016).

## Supplementary Material

Supplementary Information

Supplementary Data S1

Supplementary Data S2

## Figures and Tables

**Table 1:** Repeat elements detected in the lungfish transcriptome.

	number of elements	length occupied	percentage of sequence
Retroelements	3,918	1,408,371 bp	1.20%
SINEs:	791	103,659 bp	0.09%
LINEs:	2,668	1,178,516 bp	1.01%
L2/CR1/Rex	2,118	883120 bp	0.76%
R2/R4/NeSL	196	113911 bp	0.10%
RTE/Bov-B	10	1,598 bp	0.00%
L1/CIN4	344	179,887 bp	0.15%
LTR elements:	459	126,196 bp	0.11%
Gypsy/DIRS1	29	9,956 bp	0.01%
Retroviral	429	116,103 bp	0.10%
DNA transposons	1,902	377,257	0.32%
hobo-Activator	198	50,625	0.04%
Tc1-IS630-Pogo	1,554	293,330	0.25%
Rolling-circles	0	0	0.00%
Unclassified:	8	2,109 bp	0.00%
Total interspersed repeats:		1,787,737 bp	1.53%
Small RNA:	46	15,534 bp	0.01%
Satellites:	5	412 bp	0.00%
Simple repeats:	25,339	938,512 bp	0.80%
Low complexity:	3,166	146,658 bp	0.13%

Sequence repeats detected in the lungfish transcriptome by RepeatMasker 4.0.3, based on the vertebrate repeats database Dfam1.2. A total of 74,318 assembled transcripts were scanned, accounting for a total length of 116,965,448 bp; overall 2,886,461 bp, accounting for 2.47% of the entire assembled transcriptome, were masked.
