# Supplementary material for: The Lungfish Transcriptome: A Glimpse into Molecular Evolution Events at the Transition from Water to Land
Source: Sci Rep. 2016 Feb 24;6:21571. doi: 10.1038/srep21571 (PMC4764851; doi:10.1038/srep21571)
Supplement: Supplementary Information [file srep21571-s1.pdf]

THE LUNGFISH TRANSCRIPTOME: A GLIMPSE INTO MOLECULAR EVOLUTION EVENTS AT THE TRANSITION FROM WATER TO LAND

Biscotti Maria Assunta; Gerdol Marco; Canapa Adriana; Forconi Mariko; Olmo Ettore; Pallavicini Alberto; Barucca Marco; Schartl Manfred.

Supporting information

7 supplementary tables, 10 supplementary figures.

Supplementary Table S1: Lungfish tissues used for RNA-seq analysis.

Sample	Tissue	Sex	Specimen	RIN	SRA accession
BM	Brain	♂	1	7.9	SRX1016233
BF	Brain	♀	5	8.1	SRX1016234
LM	Liver	♂	1	7.4	SRX1016235
LF	Liver	♀	5	8.7	SRX1016236
GM1	Gonad	♂	1- immature	9.0	SRX1016237
GM6	Gonad	♂	6- mature	7.4	SRX1016238
GF2	Gonad	♀	2	9.1	SRX1016239
GF3	Gonad	♀	3	8.1	SRX1016240
GF4	Gonad	♀	4	8.3	SRX1016241

B=brain; L=liver; G=gonad; M=male; F=female. RIN=RNA Integrity Number of each sample subjected to RNA-seq. SRA=Sequence Read Archive. SRA and accession ID of the raw sequence data deposited at the NCBI database (BioProject: PRJNA164839).

Supplementary Table S2: Trimming report.

Trimming report	BM	BF	LM	LF	GM1	GM6	GF2	GF3	GF4	Previous work[3]
Reads before trimming	74,994,854	78,970,734	72,700,274	81,987830	71,802,500	79,383,536	69,561,692	74,452,106	74,124,556	142,055,980
Reads kept after trimming	74,985,223	78,954,858	72,691,053	81,696,353	71,758,237	79,362,342	69,519,358	74,427,748	74,100,133	137,287,342
Percentage of discarded reads	0.01	0.02	0.01	0.02	0.06	0.03	0.06	0.03%	0.03	3.36
Average read length before trimming	90.0	90.0	90.0	90.0	90.0	90.0	90.0	90.0	90.0	76.0
Average read length after trimming	90.0	90.0	90.0	90.0	90.0	90.0	90.0	90.0	90.0	75.6

B=brain; L=liver; G=gonad; M=male; F=female.

Supplementary Table S3: Assembly and annotation statistics.

**Assembly statistics**	
Total number of high-quality reads assembled	814,782,647
Number of contigs created	177,760
Number of base pairs in contigs	167,604,061
Average length (bp)	943
Maximum length (bp)	21,803
N75	585
N50	1,781
N25	3,907
Longest contig (bp)	21,803

N25, N50, and N75 are defined as the length of the longest contig such that all contigs of at least that length compose at least 25, 50, and 75% of the bases of the assembly, respectively.

Supplementary Table S4: Over-represented protein domains in the lungfish transcriptome.

**InterPro ID**	**Name**	***Protopterus annectens***	Grubbs oulier test vs. Actinopterygii	Grubbs oulier test vs.Tetrapoda	*Danio rerio*	*Astyanax mexicanus*	*Lepisosteus oculatus*	*Gasterosteus aculeatus*	*Takifugu rubripes*	*Tetraodon nigroviridis*	*Oryzias latipes*	*Oreochromis niloticus*	*Oncorhynchus mykiss*	*Xenopus tropicalis*	*Anolis carolinensis*	*Pelodiscus sinensis*	*Gallus gallus*	*Taeniopygia guttata*	*Homo sapiens*	*Mus musculus*	*Ornithorhynchus anatinus*	*Monodelphis domestica*
IPR000477	Reverse transcriptase domain	724 (588)	P < 0.001	NS	43	24	66	7	35	26	61	31	259	28	132	555	35	30	55	25	17	235
IPR023109	Integrase/recombinase, N-terminal	380 (273)	P < 0.001	P < 0.01	2	0	1	2	0	1	1	0	0	159	32	2	0	0	0	1	0	0
IPR010998	Integrase, Lambda-type, N-terminal	345 (250)	P < 0.001	P < 0.016	2	0	1	2	0	1	1	0	0	158	33	2	0	0	0	1	0	0
IPR005135	Endonuclease/exonuclease/phosphatase	208 (179)	P = 0.037	NS	113	41	75	53	85	90	93	50	113	203	196	197	27	29	140	97	32	217
IPR001909	Krüppel-associated box	166 (156)	P < 0.001	NS	0	0	1	0	0	0	0	0	0	52	145	131	29	2	1,486	896	38	475
IPR027299	GIY-YIG domain	153 (147)	P < 0.001	P < 0.001	0	0	0	0	0	0	0	0	0	0	0	3	0	0	0	0	0	0
IPR000305	GIY-YIG nuclease superfamily	132 (127)	P < 0.001	P < 0.001	1	1	1	2	5	3	3	0	1	1	1	2	0	0	6	7	0	2
IPR013762	Integrase-like, catalytic core	122 (57)	P < 0.001	P < 0.001	8	0	3	2	0	1	1	0	0	1	0	0	0	2	0	1	0	0
IPR004107	Integrase, SAM-like, N-terminal	84 (59)	P < 0.001	P =0.036	1	0	0	0	0	0	0	0	0	43	7	1	0	0	0	1	0	0
IPR011010	DNA breaking-rejoining enzyme, catalytic core	82 (34)	P < 0.001	P < 0.001	11	3	6	5	9	8	4	3	6	7	3	3	5	4	15	7	3	4
IPR002041	Ran GTPase	77 (59)	P < 0.001	P < 0.001	1	1	2	2	1	1	1	1	6	3	1	1	1	3	10	6	1	2

IPR003595	Protein-tyrosine phosphatase, catalytic	56 (51)	P < 0.001	P < 0.001	3	1	1	0	0	3	1	0	3	2	1	1	0	1	18	9	1	0
IPR003286	RNA-directed DNA polymerase, eukaryota	39 (29)	P < 0.001	NS	0	0	0	0	0	0	1	0	3	0	1	102	5	0	0	0	0	0
IPR006687	Small GTPase superfamily, SAR1-type	27 (19)	P < 0.001	P = NS	4	2	2	4	3	2	3	3	6	2	2	2	3	3	15	6	2	3

Over-represented InterPro protein domains detected by comparing 9 Actinopterygii and 9 tetrapod genomes. InterPro annotations for each species were retrieved from the InterPro: protein sequence analysis and classification database (http://www.ebi.ac.uk/interpro/). A p-value < 0.05 in a Grubbs test for outliers was considered a significant indication of expansion of protein families characterized by a given InterPro domain. For *P. annectens*, both the raw number of *de novo* assembled contigs and the number of non-redundant contigs based on a CD-HIT 75 % identity threshold (in brackets) are indicated. Grubbs test was performed using non-redundant sequence count. NS = not significant.

Supplementary Table S5 : Rate of molecular evolution.

Ingroup1	Ingroup2	Outgroup	Identical	Divergent	Ingroup1_specific	Ingroup2 specific	Outgroup specific	CHI^2_test	p-value	Slow
M. musculus	*P. annectens*	*C. milii*	46553	2637	3648	3158	3905	35.28	0.00000	*P. annectens*
M. musculus	*P. annectens*	*L. erinacea*	46351	2763	3657	3023	4107	60.17	0.00000	*P. annectens*
M. musculus	*P. annectens*	*S. canicula*	46603	2576	3712	3155	3855	45.18	0.00000	*P. annectens*
L. africana	*P. annectens*	*C. milii*	46473	2600	3716	3167	3933	43.79	0.00000	*P. annectens*
L. africana	*P. annectens*	*L. erinacea*	46257	2700	3739	3044	4149	71.21	0.00000	*P. annectens*
L. africana	*P. annectens*	*S. canicula*	46498	2542	3805	3136	3908	64.48	0.00000	*P. annectens*
M. domestica	*P. annectens*	*C. milii*	46654	2603	3547	3161	3936	22.21	0.00000	*P. annectens*
M. domestica	*P. annectens*	*L. erinacea*	46457	2708	3551	3052	4133	37.71	0.00000	*P. annectens*
M. domestica	*P. annectens*	*S. canicula*	46710	2563	3605	3143	3880	31.63	0.00000	*P. annectens*
P. sinensis	*P. annectens*	*C. milii*	47102	2419	3095	3153	4127	0.54	0.46309	NS
P. sinensis	*P. annectens*	*L. erinacea*	46895	2497	3109	3079	4334	0.15	0.70293	NS
P. sinensis	*P. annectens*	*S. canicula*	47179	2400	3132	3135	4050	0.00	0.96977	NS
A. carolinensis	*P. annectens*	*C. milii*	46518	2560	3683	3090	4050	51.92	0.00000	*P. annectens*
A. carolinensis	*P. annectens*	*L. erinacea*	46373	2653	3635	3045	4195	52.11	0.00000	*P. annectens*
A. carolinensis	*P. annectens*	*S. canicula*	46666	2561	3649	3123	3902	40.86	0.00000	*P. annectens*
G. gallus	*P. annectens*	*C. milii*	47144	2425	3057	3157	4118	1.61	0.20459	NS
G. gallus	*P. annectens*	*L. erinacea*	46954	2530	3054	3055	4308	0.00	0.98979	NS
G. gallus	*P. annectens*	*S. canicula*	47251	2390	3064	3185	4011	2.34	0.12585	NS
X. tropicalis	*P. annectens*	*C. milii*	45916	2677	4284	3113	3910	185.38	0.00000	*P. annectens*
X. tropicalis	*P. annectens*	*L. erinacea*	45739	2783	4268	3023	4087	212.59	0.00000	*P. annectens*
X. tropicalis	*P. annectens*	*S. canicula*	45983	2663	4331	3080	3843	211.17	0.00000	*P. annectens*
H. chinensis	*P. annectens*	*C. milii*	47011	2421	3190	3163	4116	0.11	0.73480	NS
H. chinensis	*P. annectens*	*L. erinacea*	46844	2505	3164	3105	4283	0.56	0.45617	NS
H. chinensis	*P. annectens*	*S. canicula*	47140	2417	3175	3182	3987	0.01	0.93004	NS
L. chalumnae	*P. annectens*	*C. milii*	47008	2265	3193	3290	4145	1.45	0.22831	NS
L. chalumnae	*P. annectens*	*L. erinacea*	46820	2392	3188	3168	4333	0.06	0.80192	NS

L. chalumnae	*P. annectens*	*S. canicula*	47135	2277	3180	3291	4018	1.90	0.16763	NS
D. rerio	*P. annectens*	*C. milii*	45875	2883	4326	3156	3661	182.96	0.0000	*P. annectens*
D. rerio	*P. annectens*	*L. erinacea*	45667	2927	4341	3097	3867	208.06	0.0000	*P. annectens*
D. rerio	*P. annectens*	*S. canicula*	45946	2804	4369	3192	3590	183.22	0.0000	*P. annectens*
T. nigroviridis	*P. annectens*	*C. milii*	44942	3096	5252	3044	3560	587.66	0.00000	*P. annectens*
T. nigroviridis	*P. annectens*	*L. erinacea*	44812	3214	5189	2989	3690	591.83	0.00000	*P. annectens*
T. nigroviridis	*P. annectens*	*S. canicula*	45032	3065	5276	3051	3470	594.53	0.00000	*P. annectens*
L. oculatus	*P. annectens*	*C. milii*	46686	2538	3515	3346	3815	5.16	0.04132	*P. annectens*
L. oculatus	*P. annectens*	*L. erinacea*	46450	2595	3558	3246	4051	14.31	0.00016	*P. annectens*
L. oculatus	*P. annectens*	*S. canicula*	46779	2513	3535	3351	3722	4.92	0.02660	*P. annectens*
C. milii	*P. annectens*	*P. marinus*	41473	3895	2856	2949	8727	1.49	0.22223	NS
L. erinacea	*P. annectens*	*P. marinus*	41362	4022	2967	2904	8645	0.68	0.41096	NS
S. canicula	*P. annectens*	*P. marinus*	41531	3861	2798	2927	8783	2.91	0.08821	NS

NS= not significant

Supplementary Table S6: ω ratio of genes involved in purine catabolism between *P. annectens* and other vertebrate sequences.

	UOX	*HIUase*	*PRHOXNB*	*ALN*	*ALC*
D. rerio	0.05	0.11	0.07	0.09	0.07
T. rubripes	0.04	0.14	0.11	0.11	0.11
L. menadoensis	0.07	0.12	0.07	0.12	0.14
X. tropicalis	0.06	0.16	0.09	0.10	0.11
O. anatinus	0.06	0.12	0.11	0.13	0.10
M. musculus	0.06	0.10	0.12	-	0.09

Supplementary Table S7: The 30 most abundant InterPro domains detected by InterProScan in the *de novo* assembled lungfish transcriptome.

InterPro ID	Description	No. of contigs detected
IPR027417	P-loop containing nucleoside triphosphate hydrolase	919
IPR007087	Zinc finger, C2H2	787
IPR013783	Immunoglobulin-like fold	767
IPR015880	Zinc finger, C2H2-like	730
IPR000477	Reverse transcriptase domain	724
IPR013087	Zinc finger C2H2-type/integrase DNA-binding domain	702
IPR011009	Protein kinase-like domain	523
IPR007110	Immunoglobulin-like domain	472
IPR000719	Protein kinase domain	470
IPR013083	Zinc finger, RING/FYVE/PHD-type	462
IPR023109	Integrase/recombinase, N-terminal	380
IPR011993	Pleckstrin homology-like domain	364
IPR015943	WD40/YVTN repeat-like-containing domain	346
IPR010998	Integrase, Lambda-type, N-terminal	345
IPR016024	Armadillo-type fold	336
IPR003599	Immunoglobulin subtype	335
IPR002290	Serine/threonine/dual specificity protein kinase, catalytic domain	329
IPR017441	Protein kinase, ATP binding site	326
IPR017986	WD40-repeat-containing domain	296
IPR001841	Zinc finger, RING-type	284
IPR008271	Serine/threonine-protein kinase, active site	282
IPR017452	GPCR, rhodopsin-like, 7TM	277
IPR001680	WD40 repeat	272
IPR000276	G protein-coupled receptor, rhodopsin-like	270
IPR008985	Concanavalin A-like lectin/glucanases superfamily	270
IPR001849	Pleckstrin homology domain	242
IPR012677	Nucleotide-binding, alpha-beta plait	232
IPR011989	Armadillo-like helical	230
IPR005135	Endonuclease/exonuclease/phosphatase	207
IPR003961	Proteins matched: Fibronectin, type III	206

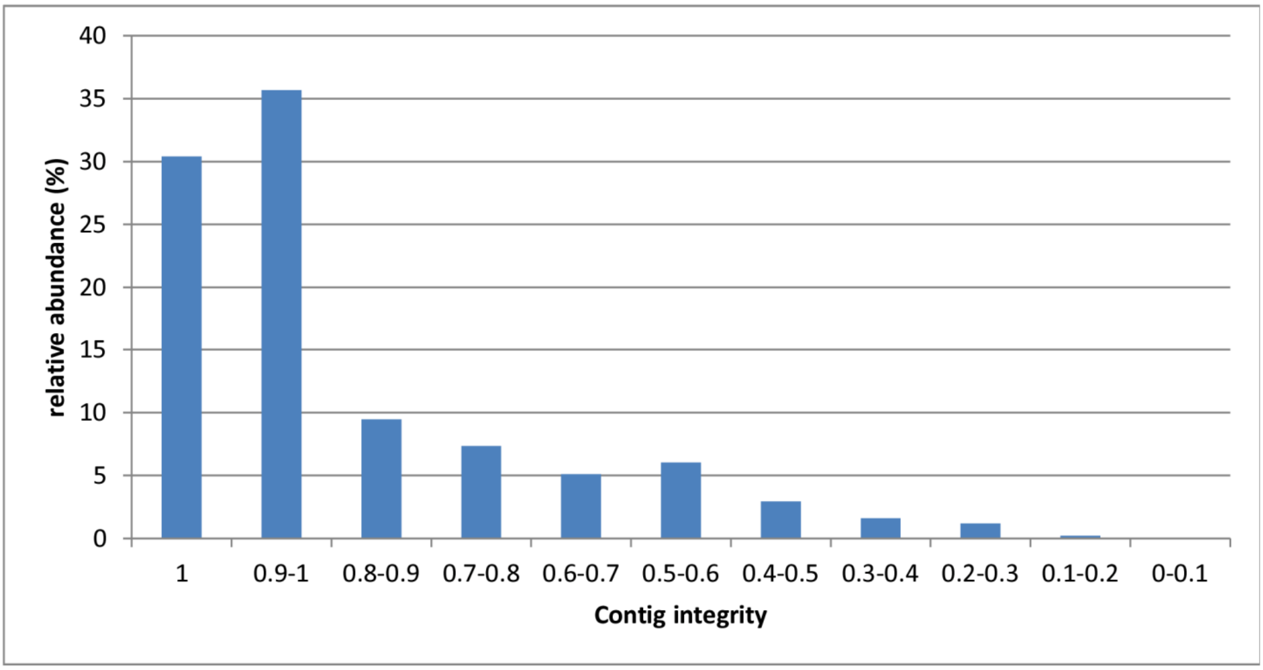

Supplementary Fig. S1: Protein-coding transcript integrity based on Ortholog Hit Ratio analysis. X axis: values approaching 1 indicate transcript integrity. Y axis: values represent the abundance of each integrity class relative to the complete transcriptome.

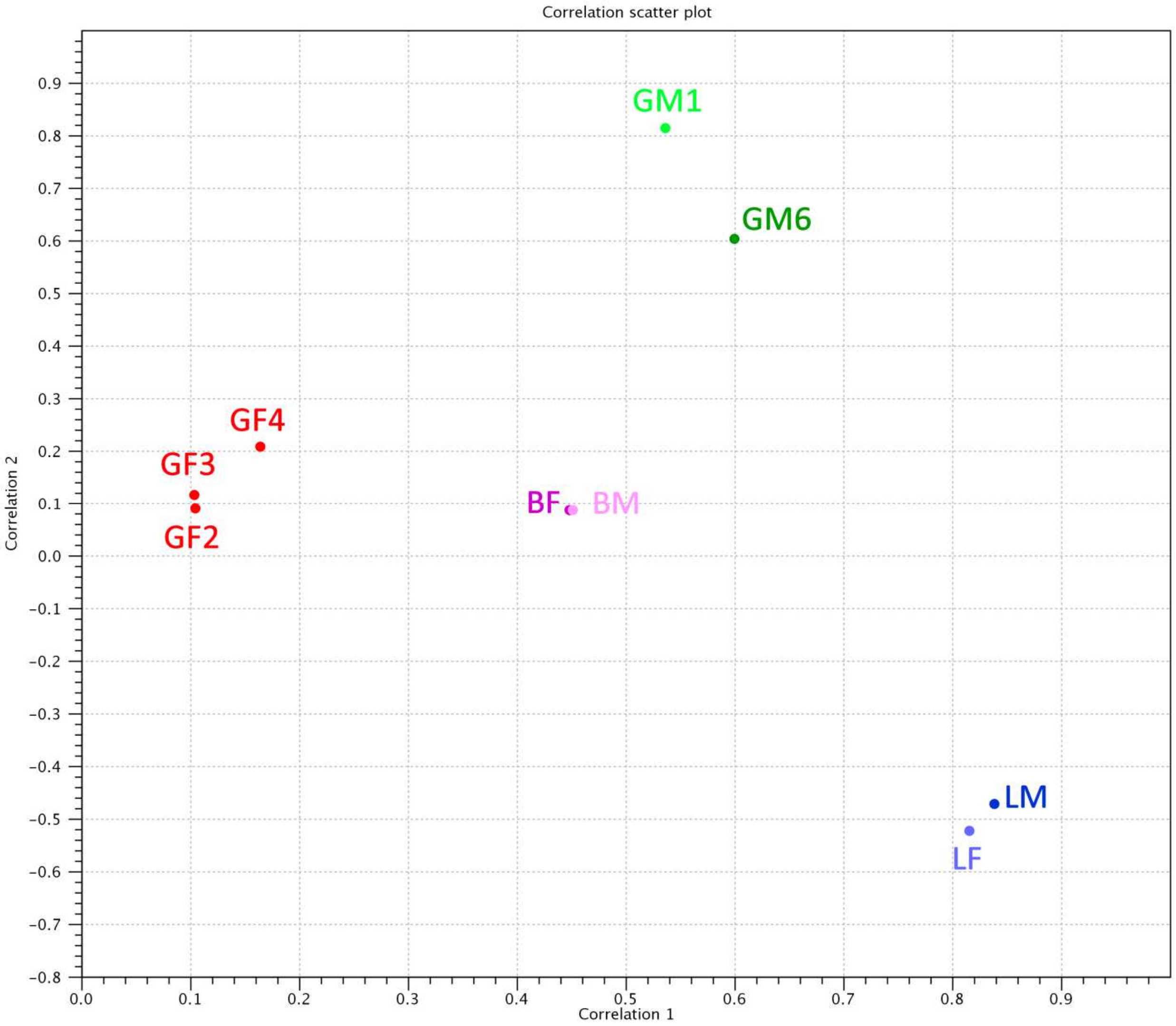

Supplementary Fig. S2: PCA showing the relationship between the gene expression profiles of the 9 lungfish samples analysed by RNA-seq. Square root-transformed TPM values of all transcripts were used to calculate the two eigenvectors, with the largest and second-largest eigenvalue plotted on the X and Y axes, respectively.

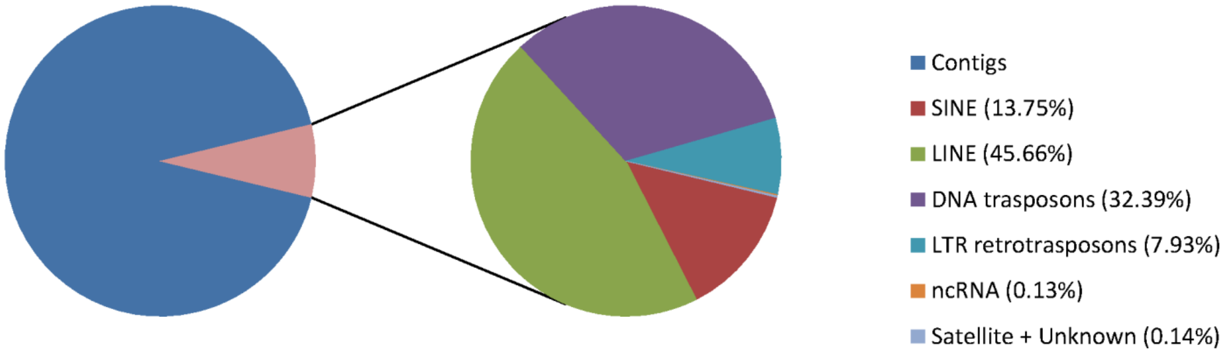

Supplementary Fig. S3: Results of RepeatMasker scan of assembled lungfish contigs. SINEs, LINEs, Class II (DNA transposons), ncRNAs (tRNAs, srpRNAs, snRNAs, 7SK RNAs), LTRs, satellite and unknown elements.

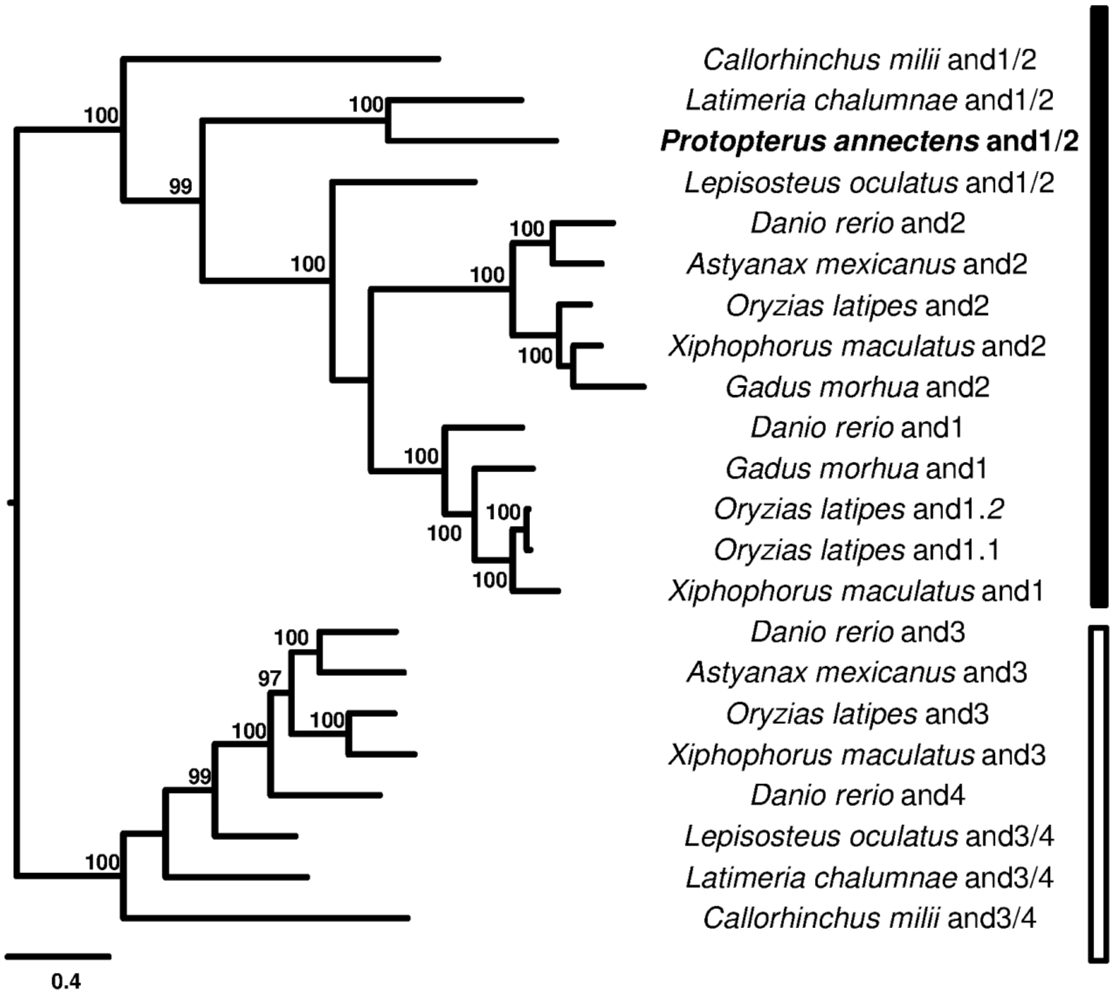

Supplementary Fig. S4: *And* gene family tree. Bayesian Inference performed with MrBayes (generations: 1,000,000; sampling: 100; substitution model: Wag; stationarity defined as the point where the average standard deviation of split frequencies reaches a value < 0.005). Black box: And1/2; white box: And3/4. Numbers on nodes represent posterior probability. The accession numbers of the aligned sequences are reported in Supplementary Data S2.

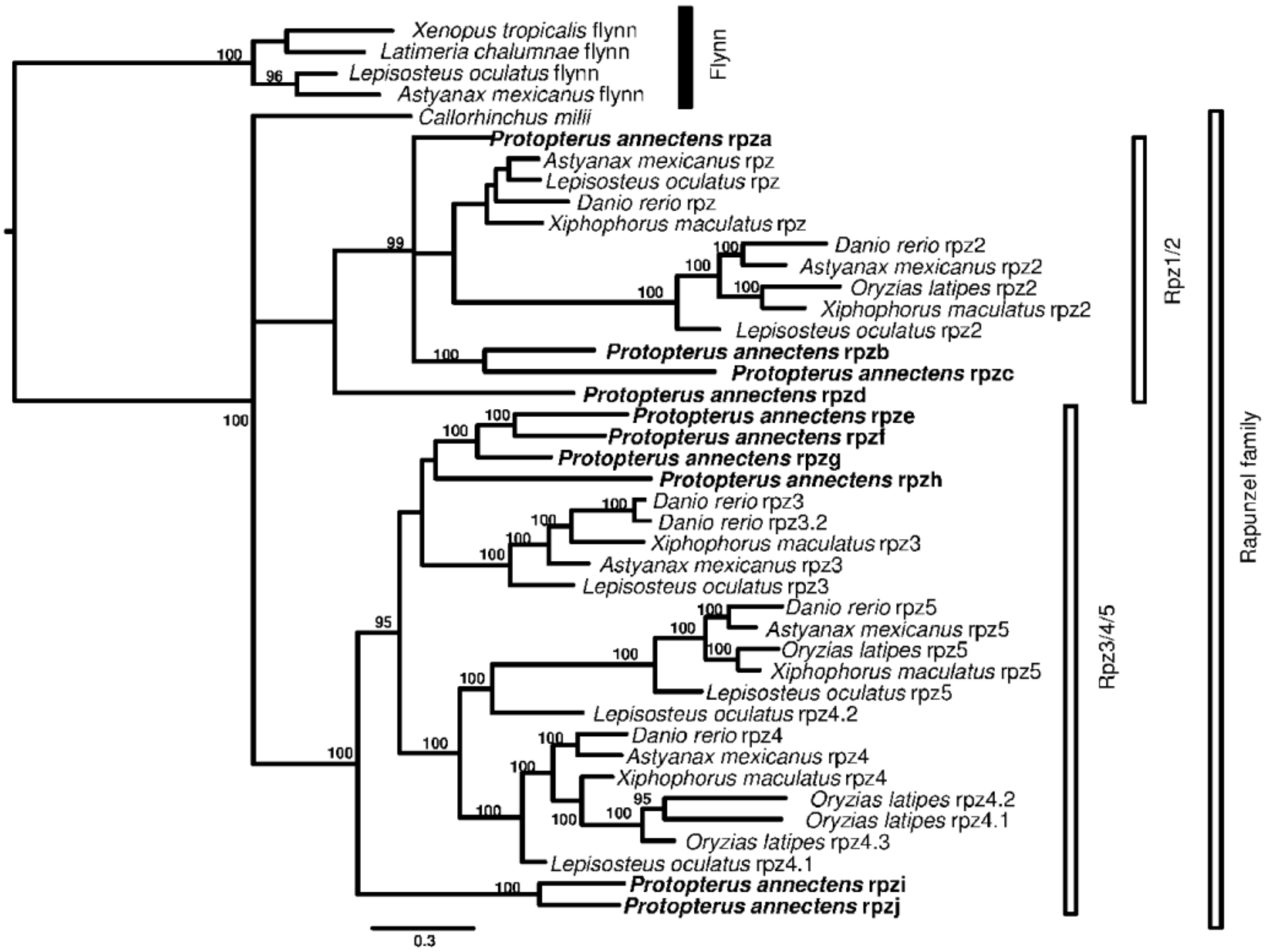

Supplementary Fig. S5: *Rpz* gene family tree. Bayesian Inference performed with MrBayes (generations: 1,000,000; sampling: 100; substitution model: Jones; stationarity defined as the point where the average standard deviation of split frequencies reaches a value < 0.006). Numbers on nodes represent posterior probability. The accession numbers of the aligned sequences are reported in Supplementary Data S2. Sequences evolutionarily related to the *rpz* family were named *'flynn'* and used as outgroups. White boxes: *rpz* family members; black box: *flynn* genes.

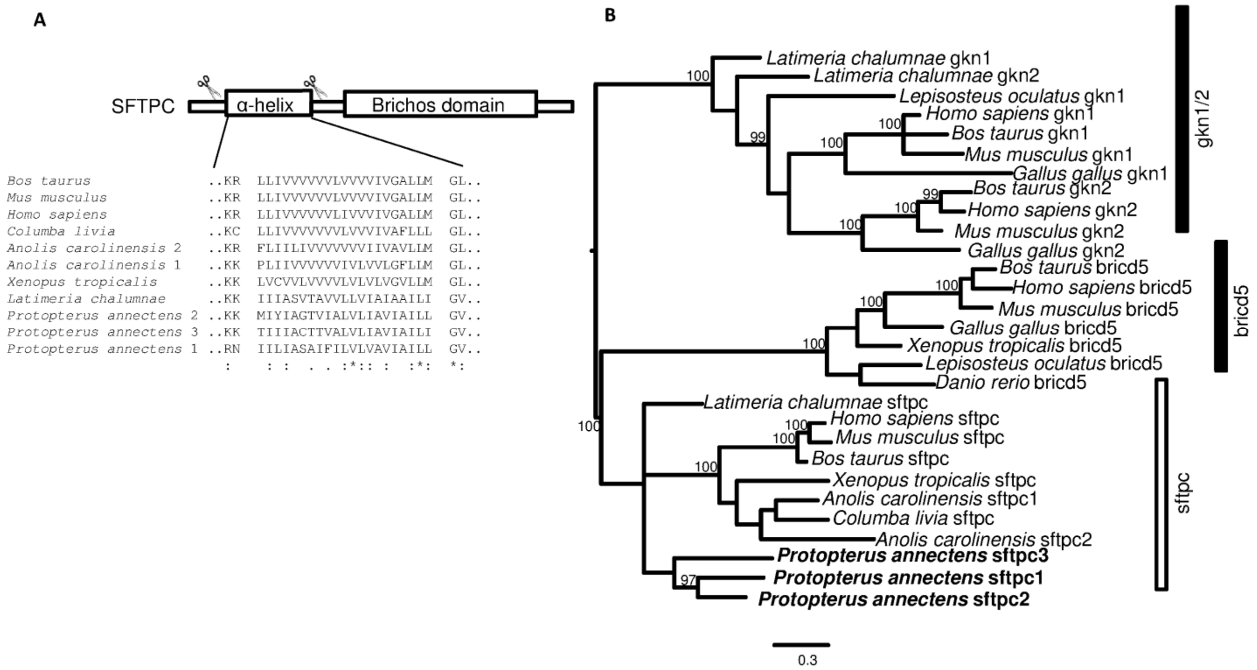

Supplementary Fig. S6: SFTPC sequence attribution. A) Multiple alignment of SFTPC α-helix region. B) Sftpc, bricd5, and gkn1/2 tree. Bayesian Inference performed with MrBayes (generations: 1,000,000; sampling: 100; substitution model: Jones + Wag; stationarity defined as the point where the average standard deviation of split frequencies reaches values < 0.005). Numbers on nodes represent posterior probability. The accession numbers of the aligned sequences are reported in Supplementary Data S2. White box: SFTPC members; black boxes: bricd5 and gkn1/2 sequences.

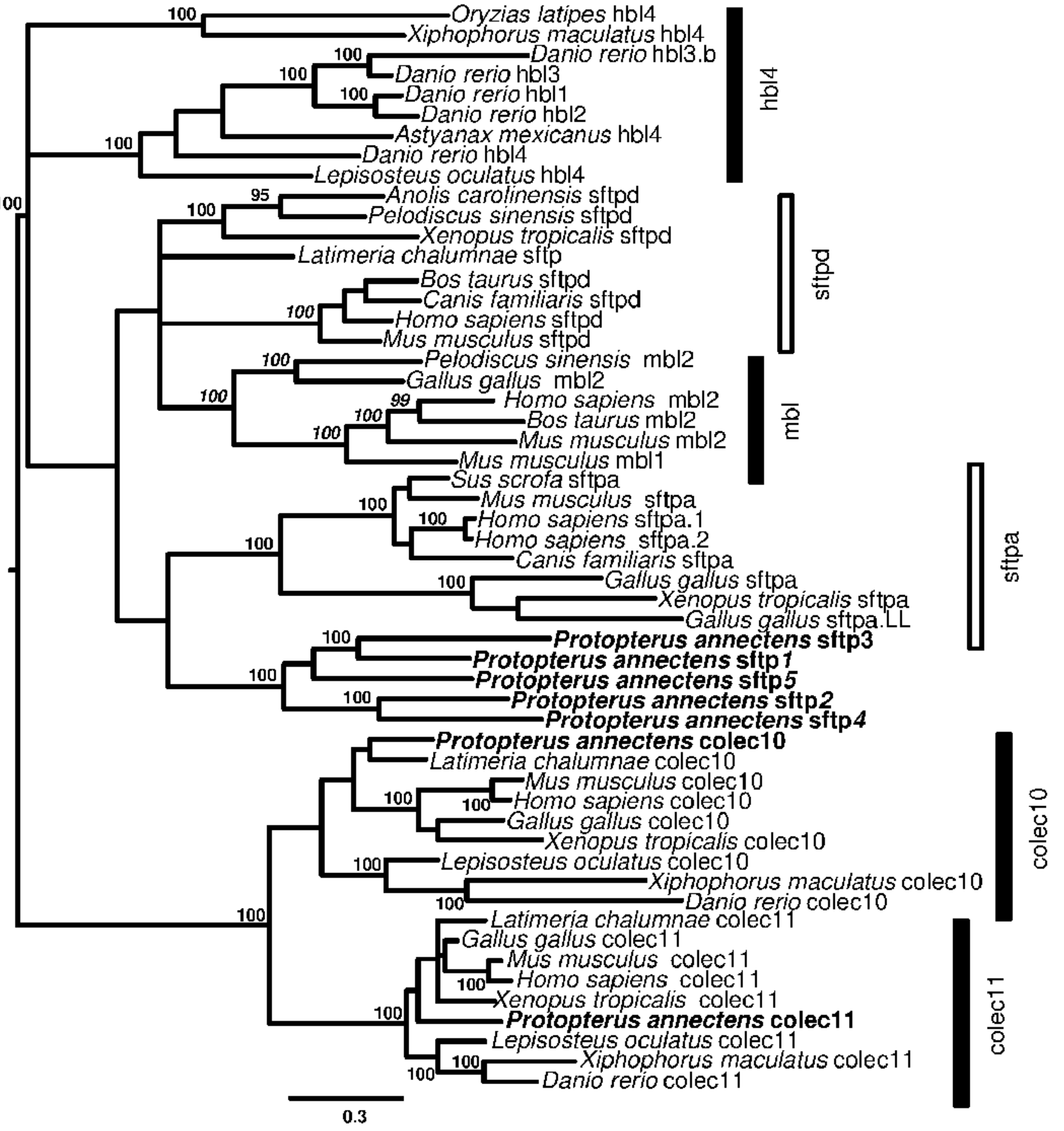

Supplementary Fig. S7: Phylogeny of SFTPA/D/MBL sequences. Bayesian Inference performed with MrBayes (generations: 5,000,000; sampling: 100; substitution model: Wag; stationarity defined as the point where the average standard deviation of split frequencies reaches a value < 0.009). Numbers on nodes represent posterior probability. The accession numbers of the aligned sequences are reported in Supplementary Data S2. White boxes: SFTPA and SFTPD members; black boxes: MBL, HBL4, and COLEC10/11 sequences.

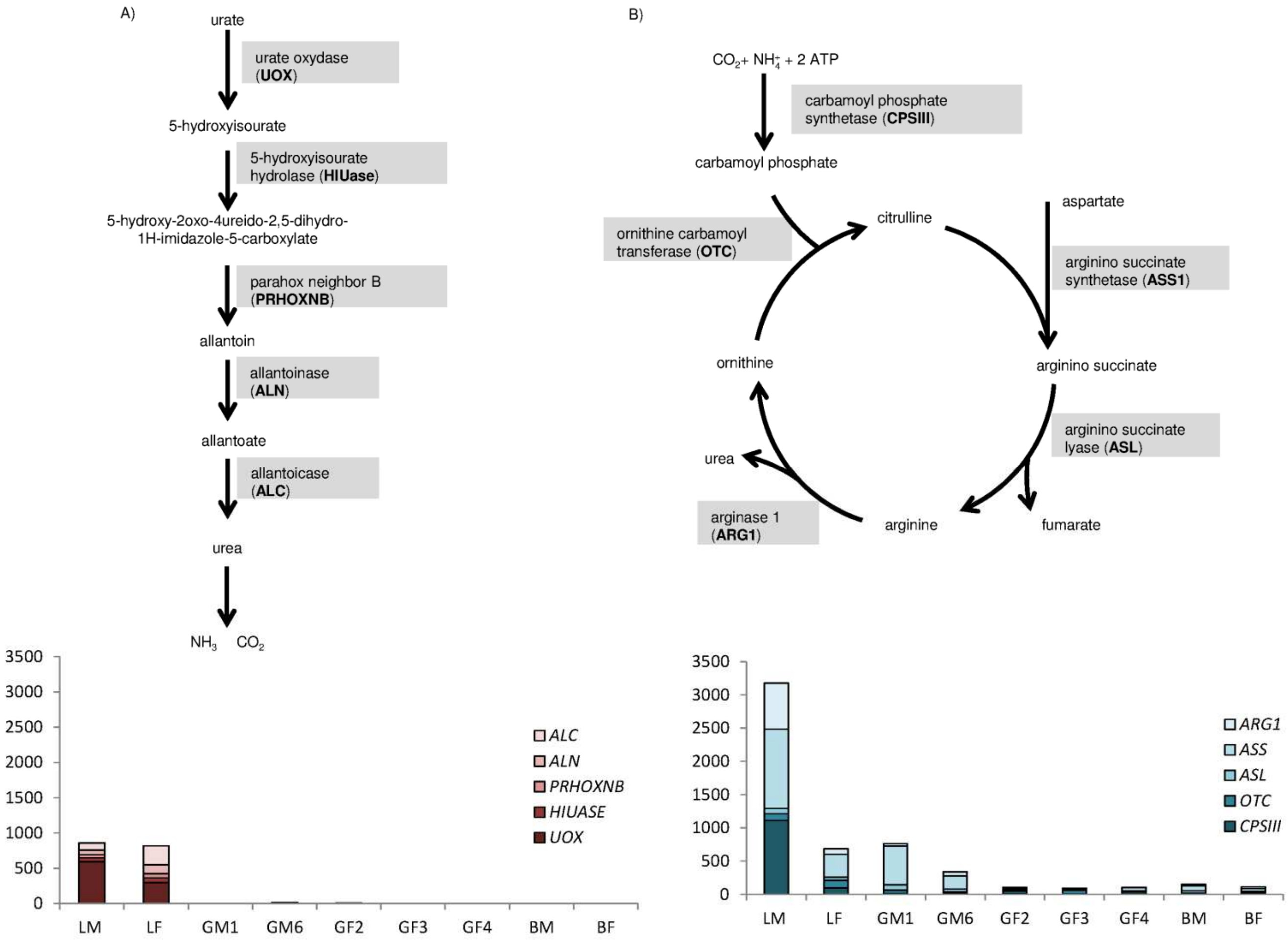

Supplementary Fig. S8: Urea pathways: A) purine catabolism; B) Urea cycle. Histograms showing the expression levels of the genes involved in each pattern in the tissues analysed. Values expressed as TPMs. Expression analysis confirmed the tissue-specificity of the genes involved in purine catabolism. Some genes involved in the urea cycle are expressed both in liver and in the male gonad.

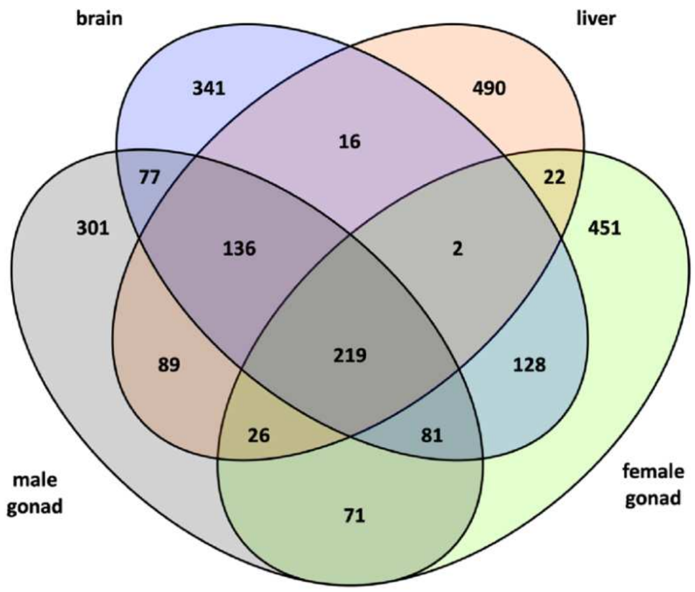

Supplementary Fig. S9: Venn diagram depicting the overlap of tissue transcriptomes assessed in the 1,000 most highly expressed transcripts in each tissue type. Whereas over 200 transcripts, serving fundamental housekeeping functions, are shared, female gonad and liver tissue appear to express a higher number of tissue-specific genes.

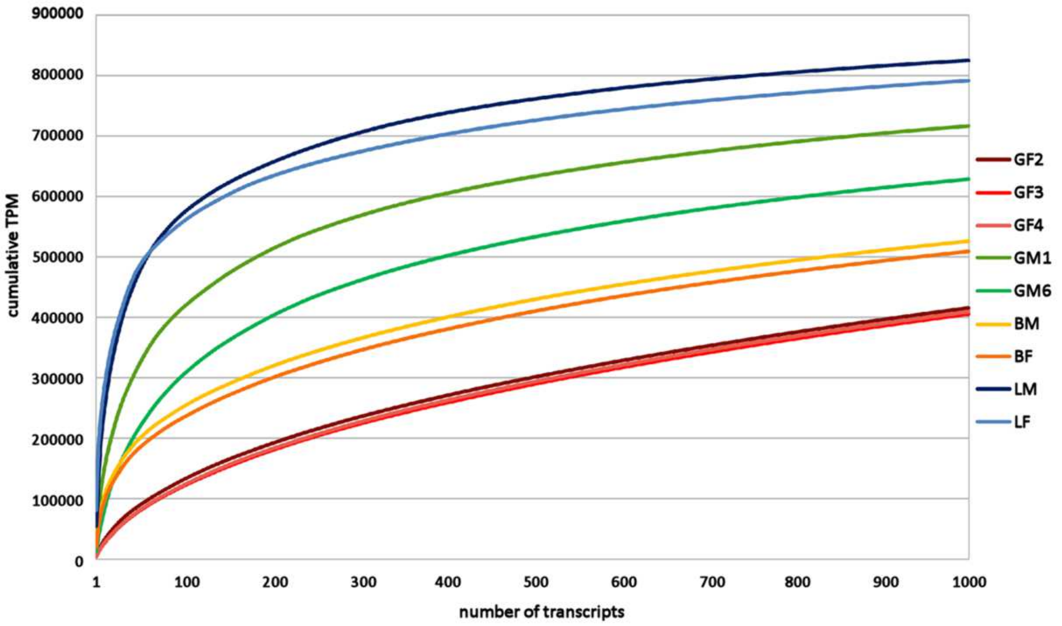

Supplementary Fig. S10: Transcriptome richness of *P. annectens* tissues. Data are shown as cumulative TPM values of the 1,000 most highly expressed transcripts in each tissue type. The 1,000 most highly expressed genes in female gonad account for 40% of all transcripts, indicating its richness in terms of number of genes expressed. Brain, male gonad, and liver tissue show a lower transcriptome richness.
